# SMARCD1 and Its Functional Relevance in SWI/SNF and Cancer

**DOI:** 10.3390/ijms27125336

**Published:** 2026-06-12

**Authors:** Jerome Pere, Colin Logie

**Affiliations:** Department of Molecular Biology, Faculty of Science, Radboud Institute for Molecular Life Sciences, Radboud University, 6500 HB Nijmegen, The Netherlands

**Keywords:** SMARCD, SWI/SNF complex, chromatin remodelling, cancer

## Abstract

In vertebrates, SWI/SNF complexes, also known as BRG1/BRM-associated factor (BAF) complexes, come in three major subtypes, canonical BAF (cBAF or BAF), polybromo-associated BAF (PBAF) and non-canonical BAF (ncBAF), that are targeted to different types of chromosomal *cis*-regulatory gene expression control elements. Approximately 20% of malignancies exhibit mutations in genes coding for subunits of the SWI/SNF family of ATP-dependent chromatin remodelling complexes. SMARCD is an essential evolutionarily conserved subunit of these complexes in all eukaryotes. Whilst the integral role of SMARCD in targeting and stabilising the SWI/SNF complexes is conserved from yeast to plants to humans, the three human SMARCD paralogs display specific expression patterns underlying their functional divergence. Although, all three SMARCD paralogs exhibit context-dependent roles in cancer, acting as both tumour suppressors and oncogenes, it is SMARCD1 that appears to show the broadest oncogenic footprint across malignancies, driving proliferation, invasion and metastasis in diverse cancer types. Here we review the recent literature pertaining to the molecular and cellular roles of the mammalian SMARCD paralogs and discuss their roles in oncogenesis from those perspectives.

## 1. Introduction

Chromatin remodelling is a crucial process involving alteration of the chromatin structure to regulate gene expression, DNA replication, repair and recombination [1,2,3]. The process of chromatin remodelling is catalysed by multiple protein complex families, of which the SWItch/sucrose non-fermenting (SWI/SNF) chromatin remodelling complex family is a prominent representative that is present in all eukaryotes studied to date [4,5,6]. These complexes can be targeted by sequence-specific DNA-binding transcription factors [7] to utilise ATP to destabilise DNA-histone interactions [8], thereby repositioning nucleosomes and adjusting access to DNA at human gene promoters, enhancers and CTCF-dependent chromatin loop anchors [9,10,11,12,13].

In plants and animals, the SWI/SNF protein complex family is composed of three main subtypes, namely Brahma-associated factor (BAF), polybromo-associated BAF (PBAF) and non-canonical BAF (ncBAF) complexes. In humans, all these complexes bear one catalytic SMARCA2 (BRM) or SMARCA4 (BRG1) SNF2-type ATPase subunit, characterised by a C-terminal bromodomain and a multitude of ancillary subunits [9,14,15].

SWI/SNF complex subtype-specific subunits have important roles in stem cell pluripotency and cellular differentiation, indicating significant roles in developmental biology [10,16,17,18,19].

Additionally, SWI/SNF complexes are particularly distinguished for their roles in tumour suppression, cell differentiation and cell cycle regulation [20,21,22,23]. Notably, approximately 20% of malignancies exhibit mutations in genes encoding SWI/SNF subunits [24]. Intriguingly, both tumour suppressive and tumour promoting roles have been ascribed to SWI/SNF subunits [5,12,25]. Altogether, the roles of SWI/SNF in stem cell pluripotency and oncogenesis make it a critical focus for cancer research.

The unique roles of SWI/SNF complexes are thought to stem from their distinct subunit compositions [20,23,26]. Of these, SMARCD1/2/3 are mutually exclusive core subunits of the BAF and PBAF complexes, while the ncBAF complex appears to only harbour SMARCD1. Intriguingly, despite the general tumour-suppressive role associated with SWI/SNF complexes, the SMARCD1 subunit stands out due to its frequent oncogenic association. This motivated us to perform the extensive review of the molecular and cellular roles SMARCD subunits presented below.

## 2. Physiological Specialisation of the SMARCD Subunits

### 2.1. Comparison and Origin of the SMARCD Paralogs

**Figure 1:**
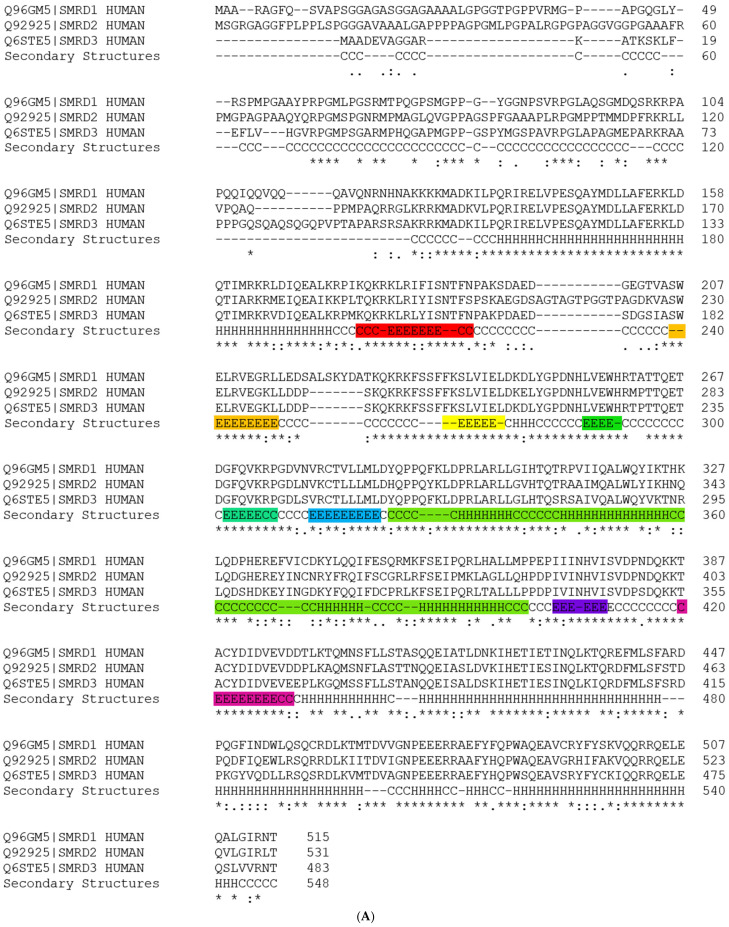

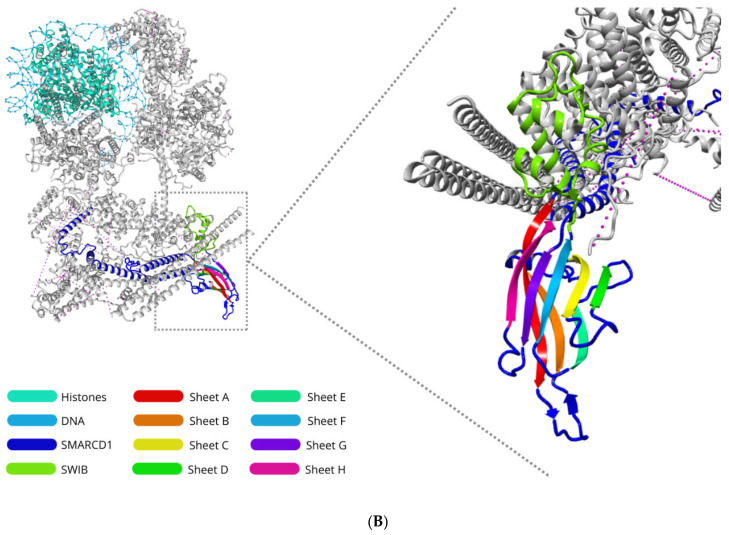
(**A**) Multiple sequence alignment of the SMARCD paralogs. Secondary structures are indicated as coiled coils (C), beta-sheets (E) and alpha helices (H). The SWIB domain that is inserted between the 6th and 7th beta-sheet (E) of the YEATS-like SWIFT domain is indicated in lime green. Fully conserved (*), strongly similar (:) and weakly similar (.) amino acid residue positions are indicated along 548 aligned positions [33]. (**B**) Structural organisation of the SMARCD1 SWIB and YEATS-like SWIFT domain within the PBAF complex. Overall view of the PBAF complex (grey) in association with the nucleosome (cyan). The dashed box indicates the enlarged region with the 8 beta-sheets that make up the SWIFT domain and the bundle of 4 alpha-helices of the SWIB domain in lime green, while the remainder of the secondary structure of SMARCD1 is rendered in dark blue.

In humans, the SMARCD (BAF60) family comprises the SMARCD1, SMARCD2 and SMARCD3 paralogs. Every SWI/SNF complex subtype described to date bears one copy of this subunit. The major isoforms of the three SMARCD paralogs share 53% sequence identity and 71% similarity, across 548 aligned positions (Figure 1A). The SMARCD family exemplifies the principle of functional divergence within a paralogous gene family. Their SWIB domains are essential for SWI/SNF complex quaternary module assembly [15,27]. Furthermore, they also share a YEATS-like SWIFT domain (Figure 1B) through which they interact directly and specifically with DNA-bound transcription factors (dbTFs), making them transcription factor co-factors [28,29,30,31,32].

To what extent the physiological functions of the SMARCD paralogs overlap in the cell types where they are co-expressed has not been analysed systematically. Nevertheless, it is evident that their physiological roles have diverged across tissues and contexts [34,35,36]. This specialisation likely serves distinct cellular and developmental needs. Mechanistically, this functional divergence is achieved by the ability of each paralog to be selectively incorporated into tissue-specific SWI/SNF complexes [37,38], thereby targeting ATP-dependent chromatin remodelling activity to distinct genomic loci in conjunction with lineage-restricted transcription factors. Indeed, each paralog governs distinct gene regulatory programs that cannot be fully substituted by the others, as revealed by acute degradation experiments in neuronal models that show both paralog-specific target gene regulation as well as compensatory protein stabilisation upon paralog loss [39]. The close grouping of SMARCD paralogs across vertebrate species suggests that SMARCD subunits perform important evolutionarily conserved biochemical functions [40,41,42,43,44].

### 2.2. SMARCD1 Expression and Physiological Functions

**Figure 2:**
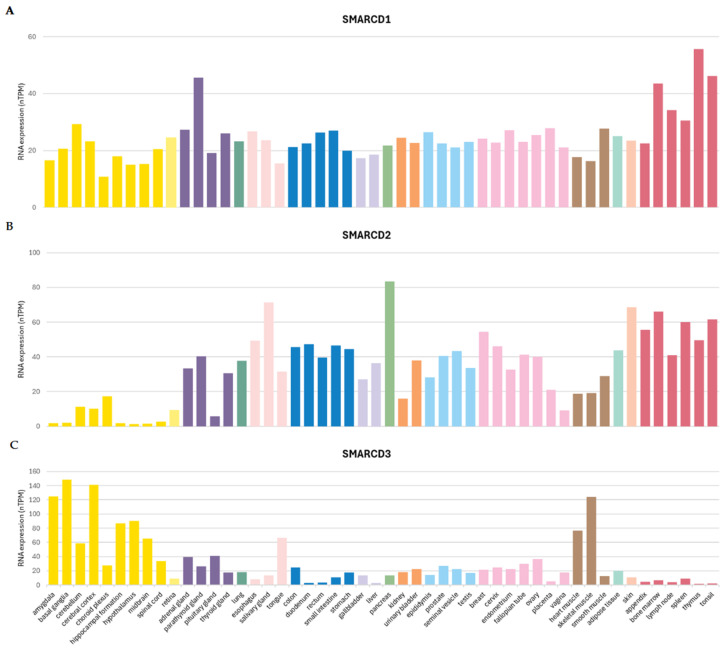
Tissue expression of the SMARCD paralogs. (**A**–**C**) RNA expression of the three human SMARCD paralogs [45]. Bars are colored by tissue category.

SMARCD1 (BAF60A) is the most extensively studied human SMARCD paralog. It is expressed at a medium to high level in most tissues [45] (Figure 2A). In the metabolic context, SMARCD1 is implicated in the regulation of hepatic fatty acid beta-oxidation and cholesterol homeostasis, interacting with the dbTFs PPARα/PGC-1α and CAR [46,47,48]. SMARCD1 also plays a role in integrating circadian signals and metabolic programs, where its deficiency can lead to obesity in mice [48,49,50]. In vascular smooth muscle cells (VSMCs) SMARCD1 and RORα directly activate the transcription of key clock genes Bmal1, Clock and Dbp, mediated by transcriptional co-activator PGC-1α (Ppargc1a). Knockdown of SMARCD1 showed disrupted rhythmic expression of these genes. SMARCD1 is also known to play crucial parts in muscle tissue development. During embryogenesis, SMARCD1 is negatively regulated by miR-133 and miR-1/206 in muscle. Furthermore, in the heart it is essential for the specific development of the anterior heart field [51,52]. Moreover, SMARCD1 is necessary for maintaining proper outflow tract septation and its deficiency in mice leads to cardiac malformations, including a shortened outflow tract, hypoplastic right ventricle and a single ventricle [51]. In skeletal muscle, SMARCD1 contributes to the overall gene regulatory network, potentially through interactions with transcription factors and signalling pathways involved in myogenesis and regeneration [37,53]. In melanocytes, MITF recruits SMARCD1 to melanocyte-specific promoters, which in turn enables BRG1 binding and chromatin remodelling required for melanin synthesis gene expression [54], with SMARCD1 additionally associating with SOX10 in this lineage [55]. In embryonic stem cells, SMARCD1 is preferentially associated with chromatin, where it directly binds KLF4 and modulates the H3K4me3/H3K27me3 balance at bivalent developmental gene loci, with its depletion leading to increased KLF4 expression and enhanced endodermal differentiation [56]. Consistent with this, ncBAF, which exclusively incorporates SMARCD1 among the BAF60 paralogs, co-localises with KLF4 at promoters in ESCs [10]. In vascular smooth muscle cells, SMARCD1 is upregulated in conditions such as abdominal aortic aneurysm, promoting inflammation and extracellular matrix degradation by recruiting BRG1 to NF-κB target genes [57]. In neurons, SMARCD1 is necessary for context-dependent gene expression involved in neural functioning and development [58,59], with mutations causing Coffin–Siris syndrome [60], a syndrome that shares many features with cohesinopathies [61,62]. This draws a broader picture where SMARCD1 perturbation is consistently pathological, but the nature of the resulting pathology is dictated by cellular context.

### 2.3. SMARCD2 Expression and Physiological Functions

SMARCD2 (BAF60B) mRNA expression is also rather non-specific, except for a lower abundance in brain regions [45] (Figure 2B). SMARCD2 plays a non-redundant role in granulopoiesis, a function proposed to be mediated by its coiled-coil 1 and SWIB domains, as overexpression of SMARCD1 fails to rescue SMARCD2-deficient cells, despite their high sequence similarity [63]. Loss-of-function mutations in SMARCD2 are directly linked to neutrophil-specific granule deficiency, resulting in neutropenia, recurrent infections and developmental abnormalities [64,65], and its absence leads to significant transcriptional alterations affecting granule protein expression and neutrophil maturation [63,64,66]. Mechanistically, the coiled-coil 1 and SWIB domains of SMARCD2 are required for the recruitment of CEBPE to promoters of neutrophilic secondary granule genes [63]. Goljanek-Whysall et al. (2014) found that, during embryogenesis, SMARCD2 is negatively regulated by miR-133 and miR-1/206 in muscle tissue [37]. In the metabolic context, the role of SMARCD2 is less defined, but its involvement in regulating immune responses may indirectly affect energy metabolism and the development of metabolic disorders [67].

### 2.4. SMARCD3 Expression and Physiological Functions

SMARCD3 (BAF60C) is detected in fewer tissues, with enhanced mRNA expression in brain regions, skeletal muscle and heart muscle [45] (Figure 2C). Studies on Smarcd3 knockout mice revealed essential roles in heart development [51], highlighting the tissue specificity and a certain level of non-redundant functionality of this SMARCD family member. The mutant mice exhibit disruptions in cardiac morphogenesis and myogenesis [37,51]. During myogenesis, microRNAs promote SMARCD3 incorporation into SWI/SNF complexes by suppressing the SMARCD1 and SMARCD2 paralogs [37]. SMARCD3 is enriched in cardiac progenitor cells and its expression is dynamically regulated during differentiation [68]. SMARCD3 bridges the cardiac transcription factors GATA4 and TBX5 to the BRG1 ATPase, an interaction necessary and sufficient to reprogram non-cardiac mesoderm towards a cardiomyocyte fate in vivo [69]. Additionally, it mediates interactions between BRG1 and NKX2-5, TBX5 and GATA4 during cardiac morphogenesis, with dosage of this complex being critical for normal heart development [70]. In skeletal muscle, an analogous mechanism operates where SMARCD3 was reported to interact directly with MyoD on muscle gene regulatory elements [71]. In the metabolic context, SMARCD3 is implicated in regulating skeletal muscle glycolytic metabolism and glucose homeostasis by interacting with the Six4 homeobox dbTF to promote Deptor expression and influence AKT signalling [28,72,73]. It also plays a role in insulin-induced lipogenesis in the liver [74].

## 3. SWI/SNF Complex Assembly and Regulation

### 3.1. SMARCD Paralogs Are Core Architectural SWI/SNF Subunits

**Figure 3:**
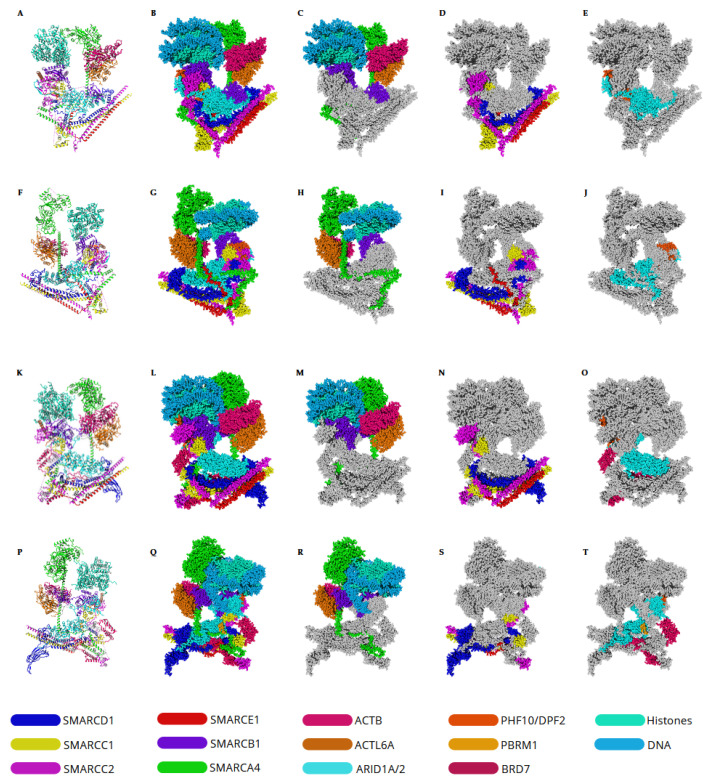
Architecture of the human nucleosome-bound BAF (**A**–**J**) and PBAF (**K**–**T**) complexes, based on the structures determined by He et al. (2020) (PDB: 6LTJ) [27] and Wang et al. (2022) (PDB: 7Y8R) [75], respectively. Front (**A**–**E**,**K**–**O)** and back (**F**–**J**,**P**–**T**) views are shown in ribbon representation (**A**,**F**,**K**,**P**) and surface representation (**B**,**G**,**L**,**Q**). Subsequent panels highlight specific subunit combinations: SMARCB1, SMARCA4, ACTB, ACTL6A, DNA and histones (**C**,**H**,**M**,**R**), structural scaffold subunits SMARCD1, SMARCC1, SMARCC2, SMARCE1 (**D**,**I**,**N**,**S**) and complex-specific targeting subunits ARID1A/2, DPF2, BRD7, PHF10 and PBRM1 (**E**,**J**,**O**,**T**). Colour coding for individual subunits is indicated in the legend. These images are original visualisations of the indicated PDB files using YASARA software (version 25.9.17) [77].

The SWI/SNF complexes share a common structural foundation involving SMARCD subunits [15,75]. SMARCD subunits directly interact with the CC domain of the SMARCC1/2 homo/heterodimer through their SWIB domain, forming an interconnected helical bundle. This interaction stabilises the core of the complexes and is necessary for their further assembly [15,27]. Next, SMARCE1 (BAF57) is incorporated, allowing binding of one SMARCA2 or SMARCA4 SNF2-ATPase subunit. It was shown early on that the addition of SMARCB1 (hSNF5, BAF47) completes the activity of the core complex [76]. Insights from the RSC complex, a yeast cell analogue of the PBAF complex, confirm that nucleosome remodelling is performed through interactions with both faces of the remodelled nucleosome, at nucleosomal DNA superhelical turn 2 (SHL2) by the SNF2-ATPase domain and at SHL6 by the SMARCB1 homolog subunits [75] (Figure 3). The deletion of SMARCB1, however, has a minimal effect on core complex assembly. Finally, all SWI/SNF subtypes share an ARP module, formed by an ACTB and ACTL6A/B (BAF53A/B) heterodimer, connecting the ATPase and core modules by interacting with the HSA domain of the SMARCA2/4 SNF2-type ATPases. The actin-related protein module further stabilises the structure and facilitates ATP-dependent DNA translocation [15,27].

### 3.2. SMARCD and SWI/SNF Complex Subtype-Specific Architecture

#### 3.2.1. PBAF Assembly

The PBAF complex possesses a set of distinct subunits [15] (Figure 3). PBAF’s name derives from the six acetyl-lysine-binding bromodomains of the PBRM1 polybromo subunit [75,78]. Following the assembly of the conserved core, PBAF-specific architecture is established by the recruitment of ARID2. ARID2’s ARM domains adopt a superhelical conformation that serves as a rigid core around which other subunits are recruited and organised. The overall architecture created by ARID2 recruits PBAF specific subunits PHF10 (BAF45A), BRD7 (CELTIX-1) and PBRM1 (BAF180) [15,75]. These subunits aid in overall structural stability and seem to be important for genomic targeting and regulating interactions with modified nucleosomal histones and DNA [79,80]. Importantly, Wang et al. (2022) found that in PBAF the YEATS-like SWIFT domain of SMARCD1 is located 120 Å away from the nucleosome core particle, indicating that it is not directly involved in binding the nucleosome that is being remodelled by the SNF2-ATPase subunit (Figure 1) [75]. The SMARCD subunit serves as an anchor for crucial subunits like ARID2, which is essential for targeting and stabilising the complex and for recruiting additional PBAF-specific targeting subunits [79,80,81].

Furthermore, insights from the yeast RSC analogue of PBAF indicate that the SNF2 ATPase C-terminal bromodomain binds the H3K14ac-modified tail of another nucleosome, rather than of the remodelled nucleosome itself [82,83]. Thus, SWI/SNF complexes possess peripheral surfaces that sense other nucleosomes though the C-terminal bromodomain of the SNF2-ATPase catalytic subunit, as well as DNA-bound transcription factors through the SMARCD subunits’ YEATS-like SWIFT domains [31]. Exactly what roles the six bromodomains of the PBAF-specific PBRM1 subunit play remains unclear, although it is known that, in the yeast RSC4 analogous subunit, one of the RSC4 tandem bromodomains binds to an acetylated residue in its adjacent bromodomain upon GCN5 histone acetyltransferase-mediated acetylation of that bromodomain [84].

#### 3.2.2. cBAF Assembly

The transition from the conserved core to the canonical cBAF-specific architecture requires the recruitment of ARID1A or ARID1B. Otto et al. (2023) found that the subsequent inclusion of subunits after ARID1A/B (or ARID2 for PBAF) is mostly dependent on parameters such as intracellular abundance, cellular context or experimental conditions [85]. These paralogous subunits are recruited to form interfaces with the SWIB domain of the SMARCD subunit and with the SWIRM domains of the SMARCC1/2 subunits. These interfaces bridge the core and the SNF2-ATPase module, facilitating recruitment and positioning of the ATPase subunit to nucleosomal superhelical location 2 (SHL2, Figure 3) [15,27,86]. Next, one copy of the DPF1/2/3 (BAF45B/D/C) and SS18/SS18L1 (SYT/CREST) paralogs are incorporated for further stabilisation and occlusion of PBAF-specific subunit interfaces [15,87]. cBAF complexes are further characterised by the presence of one of three paralogs of the functional homologs of *S. cerevisiae* RTT102, a common subunit of the yeast RSC and SWI/SNF complexes, namely the BCL7A/B/C subunits that bridge to the actin-related common ACTB-ACT6LA/B module and appear to contact the same nucleosomal acidic patch as SMARCB1 [88,89]. Contrary to the cBAF-specific ARID1A/B and DPF1/2/3 paralogous subunits, the SS18 and BCL7 paralogs have been proposed to also assemble into ncBAF complexes [9,10,89,90,91], although the initially reported ncBAF complex purified from adenovirus E1A oncogene-immortalised HEK293 cells appeared to be devoid of BCL7 and SS18 paralogs [15].

#### 3.2.3. ncBAF Assembly

The non-canonical BAF (ncBAF) complex, also known as GBAF, is a distinct SWI/SNF complex subtype characterised by a third set of unique subunits [9,10,15]. The name GBAF derives from the incorporation of GLTSCR1 or GLTSCR1L, also known as BICRA and BICRAL, alongside the bromodomain-containing protein BRD9 [92,93]. BRD9 and BICRA/L are thought to mediate interactions with modified histones like H3K27ac and other chromatin-associated factors [94,95,96].

The ncBAF complexes described to date have a specific set of core subunits. Crucially, only the SMARCD1 paralog is incorporated into the ncBAF complex, not SMARCD2 and nor SMARCD3 [9,10,92]. Similarly, ncBAF lacks SMARCB1 and SMARCE1 subunits. Moreover, ncBAF complexes harbour the core subunit SMARCC1 but not its paralog SMARCC2. Inclusion of the ACTL6A/B and beta-actin module completes the ncBAF core complex assembly.

Following the assembly of the conserved core, ncBAF-specific architecture is achieved by recruiting BRD9 and GLTSCR1/1L instead of ARID1A/B or ARID2 [92,97]. SMARCD1 plays an important role in the ncBAF complex’s structure and function as it serves as an anchor for BRD9, which is vital for targeting and stabilising the complex [10,97]. Very recently, a BCL7 subunit was proposed to be part of the ncBAF complex, being in contact with the acidic patch of the remodelled nucleosome [89,90]. Analogous compositions have been observed in plants, where BRM complexes adopt ncBAF-like features requiring BRD subunits for assembly and stability [98].

### 3.3. Specialisation of SWI/SNF Complexes for Different Types of Cis-Regulatory Elements

Before CTCF was demonstrated to be a chromatin loop anchoring factor that functions by blocking cohesin complex-mediated chromatin loop extrusion progress in an orientation-dependent fashion [99,100], gene regulation was thought to act through some looping between gene promoters and enhancers [101]. After the initial description of topologically associated domains (TADs) [102], CTCF sites became best known for their enrichment at TAD boundaries [99] that insulate sub-mega-base chromatin domains from the influence of distal gene enhancers. More recently, it was found that vertebrate genomes can be partitioned in alternating left and right parts of TADs, which are respectively enriched in right- and left-pointing CTCF sites that encode sets of nested loops, whereby divergent CTCF site configurations mark interloop regions that operate as TAD boundaries and are enriched in short genes [13]. Intriguingly, more than 7000 human protein-coding genes bear at least one CTCF site within 2.5 kb of their transcription initiation site, indicating that gene promoter function and CTCF-dependent chromatin loop anchor function can co-occur at the same genomic elements [103].

Similarly, many gene promoters can enhance transcription from other gene promoters when they bear similar transcription factor binding motifs, indicating that many promoters can moonlight as enhancers [104], possibly by contact-independent proximity-dependent mechanisms [105]. Altogether, there would therefore appear to be three non-exclusive types of cis-regulatory elements. The first are gene promoters, which bear initiation sites for (m)RNA transcription. The second being enhancers and silencers, which can modulate the activity of promoters that are under their influence. The third being chromatin loop anchors, which synapse convergent CTCF sites that can be more than one megabase apart, bringing distal gene regions into physical proximity and confining enhancer and silencer activity within genetically encoded chromatin loop networks [13,106]. Surprisingly, a rather straightforward level of specialisation towards gene promoters, enhancers and CTCF-based chromatin loop anchors [107] appears to distinguish the roles of the three major vertebrate SWI/SNF complex subtypes.

**Figure 4:**
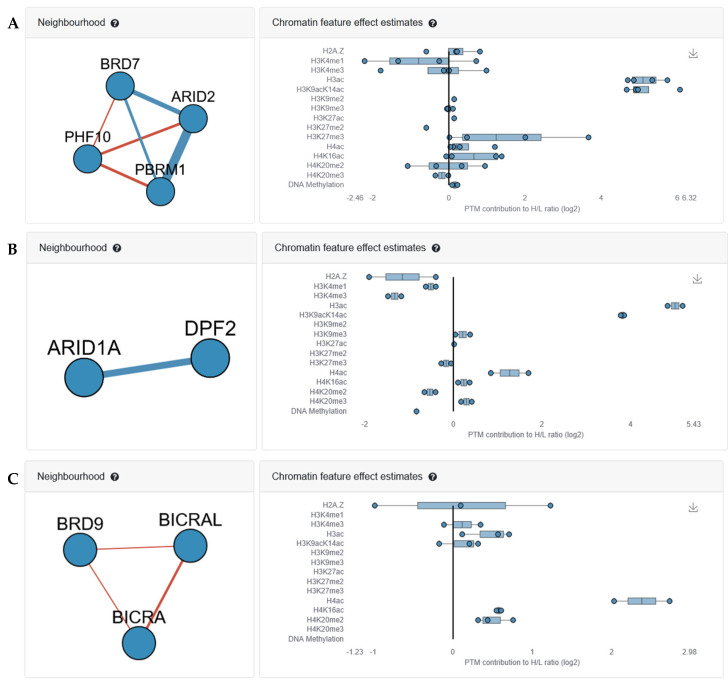
Chromatin feature associations and subunit neighbourhoods of SWI/SNF complex variants. (**A**) PBAF-specific neighbourhood. (**B**) cBAF-specific neighbourhood. (**C**) ncBAF-specific neighbourhood. The *x*-axis represents relative strength of recruitment or exclusion of the indicated SWI/SNF subunit group by histone post-translational modification, as measured by differential isotopic amino acid labelling. Data were visualised in the online MARCS database [115].

PBAF exhibits a unique preference for promoters and gene bodies [108,109]. This must be due to the combined presence of ARID2, PBRM1, BRD7 and PHF10, together with the absence of BAF and ncBAF-specific subunits, resulting in specific interactions with nucleosomal histones and other chromatin-associated factors [110,111,112]. Indeed, in the context of chromatin immunoprecipitation and programmed chromatin pull downs [113,114], the PBAF-specific ARID2, BRD7, PBRM1 and PHF10 subunits are attracted to nucleosomal histone-borne post-translational modifications that mark promoters, namely H4ac, H3ac and the canonical gene promoter mark H3K4me3 that is found at many CpG islands (Figure 4A).

The BAF complex is particularly active at enhancers and specific type-1 promoters that exhibit enhancer-like chromatin features, such as a high H3K4me1/H3K4me3 ratio and low chromatin accessibility [15,113,116,117]. cBAF’s modular subunit composition likely ensures such enhancer targeting [85,118,119]. Indeed, the BAF-specific subunits DPF2 and ARID1A appear to be attracted to H4ac and H3ac that mark enhancers and promoters but are repulsed by the promoter-specific H3K4me3 mark (Figure 4B) [113,114].

Finally, unlike cBAF and PBAF, ncBAF exhibits a preference for CTCF and cohesin-binding sites, certain enhancers and promoter regions, often co-localising with KLF4, SP5 and CTCF [10,94]. This unique localisation is attributed to its distinct subunit composition. Indeed, BICRA, BICRAL and BRD9 appear to mainly associate with H4 acetylation but not as much with H3ac as PBAF and BAF (Figure 4C) [113,114].

Since SMARCD1 appears to be the only SMARCD paralog that is incorporated into the ncBAF complexes, it may be that its unique role in cancer is in part caused through dysregulation of the ncBAF complex and therefore of the genome partitioning systems that rely on CTCF-mediated chromatin loop network dynamics.

### 3.4. SWI/SNF Complex Subunits Are Targets of Intracellular Signal Transduction

Laboratories that chart proteome-wide post-translational amino acids modifications (PTM) have documented a multitude of SWI/SNF subunit modifications [33,120,121,122]. One set of experimentally confirmed PTM concerns the ARID1A cBAF-specific subunit that is degraded in a phosphorylation and ubiquitylation-dependent fashion upon DNA damage [115,123,124] and also in multiple cancers, where it has been suggested that the consequent loss of cBAF functionality allows the cancer cells to escape a strict enforcement of their terminal cellular differentiation program, allowing the cells to adopt more stem cell-like oncogenic properties [125].

**Figure A1:**
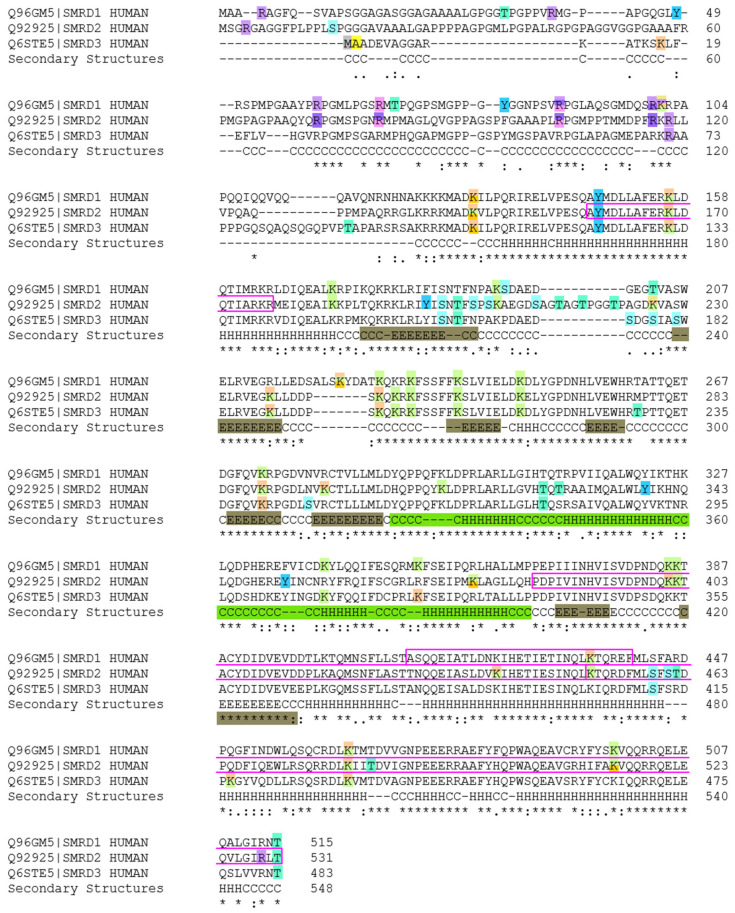
Multiple sequence alignment of the SMARCD paralogs, colour-coded with post-translational modifications, regions and domains. Colour codes are referred to in Table A1. Fully conserved residues (*), groups of strongly similar properties (:) and groups of weakly similar properties (.) are indicated.

In the case of the three SMARCD paralogs, Table A1 indicates that, of the 548 aligned amino acid positions in Figure 1A, 66 are the subject of 140 experimentally detected post-translational modifications (Appendix A Figure A1). These include arginine methylation, lysine sumoylation, ubiquitylation, methylation and acetylation, as well as serine, threonine and tyrosine phosphorylation. In some cases, such as sumoylation of lysines 153, 178 and 497, ubiquitylation of lysines 178, 261, 265, 271, 279 and 497, acetylation of lysine 153 and phosphorylation of tyrosine 169, the PTM was independently documented for each of the three human SMARCD paralogs. Intriguingly, the top predicted kinases for tyrosine 169 belong to the JAK family. These have been documented to phosphorylate histone H3Y41 in the nucleus and may therefore also target the three SMARCD paralogs [126]. However, to date, there are very few dedicated studies addressing the physiological role of SMARCD paralog modifications.

In SMARCD1, threonine at positions 37 and 83, and tyrosine at position 59 are absent in the SMARCD2 and D3 paralogs. The threonine residues are predicted substrates of the CMGC kinase superfamily; position 37 is predicted to be targeted by CDK18 and P38 paralogs (MAPK11-13) and position 83 is a predicted target for all three JNK kinases (MAPK8-10) [127]. Since P38 and JNK are canonical effectors of inflammatory stress signalling [128], phosphorylation at positions 37 and 83 may reflect SMARCD1 responsiveness to inflammatory contexts [57]. Tyrosine at position 59 is predicted to be phosphorylated by ABL1 and ABL2, which have established roles in oncogenic chromatin signalling [129,130], potentially placing SMARCD1 as a candidate effector of ABL-driven nuclear signalling. SMARCD1 serine at position 217 is predicted as a casein kinase (CK2) substrate, potentially linking SMARCD1 to the CK2-androgen receptor axis implicated in prostate cancer [131,132,133]. Finally, lysine at alignment position 256 resides within a SMARCD1-specific insertion between beta sheets B and C of its YEATS-like SWIFT domain (Figure 1B) and it is subject to ubiquitylation, sumoylation and acetylation, perhaps indicating a regulatory pathway that is unique to SMARCD1-bearing SWI/SNF complexes.

SMARCD2 threonine residues at positions 227 and 231 are located within a SMARCD2-specific sequence and they are also predicted to be substrates for the CMGC kinase superfamily [127]. More specifically, the stress-activated protein kinases (SAPKs) include JNK and various P38 paralogs (MAPK8-14). Since the JNK/P38 kinases are canonical effectors of inflammatory stress and cytokine signalling [128], SMARCD2 may perhaps act as a direct chromatin effector of inflammatory responses in the context of granulopoiesis [63,64,65]. The presence of predicted sites for CDK1 at position 231 and for ERK1 (MAPK3) at 227 might also link SMARCD2 activity to cellular proliferative status [127,134,135]. However, in the absence of experimental evidence, this remains purely speculative.

Notably, SMARCD3 is phosphorylated at coordinates 233 and 312, which are not conserved sites in the SMARCD1 and D2 paralogs. Coordinate 233 predictions are dominated by BMP type I and II receptors BMPR1A, BMPR1B, ACVR2A and ACVR2B, alongside TGF-β and activin receptors TGFBR1, ALK2, ALK4 and ALK7, all belonging to the TKL kinase group [127]. This might implicate SMARCD3 as an effector of BMP/TGF-β receptor signalling, consistent with its essential role in cardiac development [51,136,137]. Coordinate 312 is predicted to be targeted by mixed lineage kinases MLK1, MLK3 and MLK4, as well as TGFBR2, potentially extending the TKL signalling connection to a second SMARCD3-specific site [127]. Finally, coordinate 294 (T229pho) was experimentally confirmed as the phosphorylation site targeted by p38α (MAPK14) during MyoD-dependent skeletal muscle differentiation [71], making it the only SMARCD paralog PTM with a directly demonstrated physiological function to date.

Overall, it would therefore appear that studying SMARCD paralog PTMs may be a fertile future research area, both to understand basic physiology and in the context of pathologies such as cancer.

## 4. SMARCD Paralogs and Cancer

Historically, the SWI/SNF complex has been predominantly recognised for its tumour-suppressive functions [12,25]. The SMARCD family subunits, however, appear to exert context-dependent influences on cancer development and progression as they have been reported to function as both tumour suppressors and oncogenes depending on expression level, cellular context and the specific paralog involved [30,34,138,139,140]. This functional plasticity suggests that SMARCD proteins serve as flexible rheostats that translate the cellular signalling environment into specific genomic outputs [141]. Intriguingly, the current cancer-related literature is biased toward SMARCD1, with SMARCD2 and SMARCD3 being studied predominantly in tissue-restricted physiological contexts. Whether this imbalance reflects a lack of systematic investigation or a genuine SMARCD1 paralog-specific oncogenic potential remains an open question (Appendix B Table A2).

### 4.1. SMARCD SWI/SNF Subunits as Tumour Suppressors

It has been suggested that SMARCD subunits can function as tumour suppressors when their expression enforces differentiation and apoptotic programs. In glioblastoma, the characteristic downregulation of SMARCD1 partially disables these processes, where its restoration suppresses proliferation and sensitises cells to temozolomide by inhibiting the Notch1 pathway and enhancing p53-mediated apoptosis [142]. Similarly, loss of SMARCD3 was recently reported to promote cervical cancer progression [138]. These examples indicate that SMARCD subunits enable differentiation and apoptotic programs when properly expressed and regulated, with their loss disabling cellular emergency brakes against malignant transformation. Hence, SMARCD paralog-mediated tumour suppression and oncogenicity may ultimately be dictated by the activity of their upstream regulators such as sequence-specific DNA binding transcription (co)factors that recruit SWI/SNF complexes to regulatory cis-acting elements and enzymes that modify SMARCD subunits within specific cellular contexts.

### 4.2. SMARCD SWI/SNF Subunits as Oncogenes

#### 4.2.1. miRNAs as Suppressors of the Oncogenic Potential of SMARCD1

Across diverse cancers, oncogenic activity is driven by the loss of tumour-suppressive microRNAs. For the SMARCD paralogs this can result in overexpression or dysregulation through multiple convergent mechanisms. A recurrent oncogenic pathway involves the loss of tumour-suppressive microRNAs from the miR-99 family (miR-99a, miR-99b, miR-100) that collectively target SMARCD1 across prostate, breast and bladder cancers. In prostate cancer, this dysregulation links to increased PSA production, altered radiation responses via the glucocorticoid receptor in cancer stem cells and mTOR pathway activation in castration-resistant disease [34,133,143,144,145,146]. Similarly, in breast cancer, miR-100-mediated suppression of SMARCD1 was reported to inhibit breast cancer stem-like cell (BCSC) self-renewal, while in bladder cancer, the loss of miR-99a-5p upregulates SMARCD1 to promote gemcitabine resistance and inhibit senescence [147,148]. Beyond the miR-99 family, promoter hypermethylation of miR-490-3p in gastric cancer was reported to increase SMARCD1 expression to promote proliferation, colony formation, invasiveness and poor survival [149]. This is consistent with the fact that genomic studies have identified SMARCD1 as a recurrent driver mutation in breast cancer and gestational choriocarcinoma [150,151,152].

#### 4.2.2. SMARCD Paralogs and WNT Signalling

Wnt signalling represents a central oncogenic axis utilised by SMARCD paralogs to drive malignancy in a paralog-specific manner. SMARCD3 promotes Wnt-mediated EMT in breast and gastric cancers. In breast cancer, it maintains Wnt5a expression and chromatin accessibility to drive EMT, with its silencing triggering mesenchymal-to-epithelial transition and its overexpression increasing CD44+/CD24- stem-like cells [153,154], whereas in gastric cancer it was reported to operate through a distinct PI3K-AKT/Wnt-mediated EMT pathway [155,156]. This Wnt regulation extends to colorectal cancer-associated fibroblasts, where SMARCD3 correlates with advanced disease and immune suppression via M2 macrophages and neutrophil infiltration [157]. These SMARCD3-mediated invasive programs further extend to paediatric brain tumours, where a neurodevelopmental Reelin-DAB1-Src signalling axis is highjacked to drive metastatic dissemination through leptomeningeal seeding [158]. Conversely, in gastric cancer stem cells (GCSCs), SMARCD1 was reported to serve as a Wnt/β-catenin regulator, sensitive to pharmacological inhibition by ibuprofen and pantoprazole [159,160].

#### 4.2.3. Immune Checkpoint Modulation and Immunosuppression

Immune checkpoint modulation and immunosuppression represent another axis of SMARCD influence. SMARCD1 facilitates immune escape by maintaining PD-L1 promoter accessibility in colorectal cancer [161] and driving CD8+ T cell exhaustion via CCL8/glycolytic gene regulation in oesophageal squamous cell carcinoma [162]. Conversely, its low expression marks immunologically “cold” states in diffuse large B-cell lymphoma [163]. In the former, PD-L1 expression is effectively suppressed by the SWI/SNF inhibitor FHT-1015. Pan-cancer analyses confirm SMARCD subunits as oncogenic biomarkers. Here, SMARCD1 overexpression signals poor prognosis in skin cutaneous melanoma [140], while SMARCD3 associates with poor outcomes across multiple malignancies, enrichment in cell cycle and p53 pathways and correlation with immunosuppressive cell infiltration and checkpoint expression, including PD-L1 and CTLA4 [164]. This prognostic utility extends to cuproptosis-related signatures in endometrial cancer [165] and risk stratification in advanced-stage neuroblastoma [166].

#### 4.2.4. Metabolic Reprogramming and Therapy Resistance

Metabolic reprogramming and therapy resistance are similarly fuelled by subunit-specific survival pathways. SMARCD1 primarily regulates the mTOR and glycolytic axes, with its activation of mTOR signalling correlating with poor survival in hepatocellular carcinoma [167,168,169] and castration-resistant prostate cancer [145], while promoting immunosuppressive lactate production via glycolytic gene regulation in oesophageal squamous cell carcinoma [162]. Consistent with this mTOR-activating role, kinase prediction analysis identifies PERK kinase as a SMARCD1-kinase, potentially linking ER stress responses to SMARCD1-driven mTOR activation [127,170,171]. These SMARCD1-driven mTOR programs represent actionable therapeutic targets, effectively inhibited by Gecko herb miRNAs in hepatocellular carcinoma [172] and through combined mTOR/SMARCD1/androgen receptor targeting in prostate cancer [145]. Distinctively, SMARCD2 functions as a metabolic responder in diffuse large B-cell lymphoma, where it is upregulated in response to metabolic dysfunction to promote lymphoma development [163]. In contrast, SMARCD3 was reported to help maintain metabolic flexibility and cell cycle progression, specifically through fatty acid oxidation in pancreatic ductal adenocarcinoma [139], by regulating the cell cycle through cyclins CCNA2, CCNB1 and cyclin-dependent kinase (CDK1) expression in ER+ breast cancer [173] and by bypassing mTOR inhibition in resistant ER+ breast cancer via MAPK/ERK upregulation [174].

#### 4.2.5. Oncogenic Gene Expression Programs

As essential co-factors for sequence-specific DNA-binding transcription factors, SMARCD subunits enable oncogenic programs across multiple malignancies. For SMARCD1 this includes the androgen receptor (AR) and HOXB13 in prostate cancer, mutant p53 in oral squamous cell carcinoma, c-Raf in acute myeloid leukaemia, HNF4A in pancreatic adenocarcinoma and the POU2F3 oncogenic program in small cell lung cancer [29,30,132,133,139,175,176]. The latter being effectively disrupted by BRD9 degraders and ATPase inhibitors [30]. Furthermore, combined ATPase and chromatin co-factor inhibition demonstrates synergistic efficacy, exemplified by dual PROTAC-mediated degradation of P300 and SWI/SNF in synovial sarcoma, which broadly disrupts SS18-SSX-driven oncogenic transcription [91]. Conversely, in cancers with complete dual SMARCA4/SMARCA2 loss, dependency shifts to alternative chromatin re-modellers, with CHD3/NuRD complex depletion triggering synthetic lethality through toxic de-repression of PARD3B and subsequent MYC pathway collapse [177]. Similarly, SMARCD2 appears to sustain the leukemic state through the maintenance of oncogenic transcriptional programs in MLL-rearranged leukaemia [178]. Genomic disruption of these TF-SMARCD interactions, as seen in rare paediatric undifferentiated carcinomas harbouring SMARCD2 p.Arg391Cys mutations within the conserved BAF interaction domain, leads to widespread transcriptional dysregulation and loss of cellular differentiation [179].

Cancer stem cell (CSC) maintenance and therapy resistance emerge as interconnected oncogenic roles for SMARCD subunits. SMARCD1 sustains CSC properties through MUC1-C/E2F1-mediated pluripotency factor expression in neuroendocrine prostate cancer [180] and Wnt/β-catenin signalling in gastric CSCs [159]. It also drives therapy resistance by promoting gemcitabine resistance and inhibiting senescence in bladder cancer [148]. Similarly, SMARCD3 expands the CSC pool by increasing CD44+/CD24- populations in breast cancer [153] and maintaining fatty acid oxidation programs in pancreatic ductal adenocarcinoma [139]. This stemness-promoting role facilitates chemotherapy resistance in ER+ breast cancer, where SMARCD3 enables a metabolic bypass that underlies a vulnerability to MEK inhibitors [174].

### 4.3. Context-Dependent SMARCD1 Function

SMARCD1 function exhibits non-linear relationships with clinical outcomes in specific contexts. In ER+/HER2-enriched breast cancer, SMARCD1 demonstrates a U-shaped survival curve where both high and low expression correlate with improved outcomes while intermediate levels associate with worse distal metastasis-free survival, indicating that tight expression control is critical for normal function [181]. In ovarian cancer, SMARCD1 function is microenvironment-dependent, where ascites exposure drives more than 2.5-fold SMARCD1 upregulation to promote migration and invasion [182], while in high-grade serous ovarian cancer, its expression is inversely correlated with tumour suppressing miR-223 [183] and is upregulated via DLEU1/miR-490-3p interaction to promote proliferation [184]. These findings demonstrate that SMARCD1 plays distinctive context-dependent roles in endocrine related cancers.

The SMARCD1-specific oncogenic profile may reflect its predominant incorporation into ncBAF complexes, as detailed in Section 3.2.3 and Section 3.3 In healthy cells, proper SMARCD1 expression within ncBAF complexes preserves chromatin boundaries alongside its roles in cBAF-mediated enhancer activation and PBAF-mediated cell cycle regulator expression. However, cancer-associated mutations likely alter this delicate balance. We speculate that SMARCD1 dysregulation-mediated oncogenic transformation occurs through three interconnected mechanisms: loss of PBAF tumour-suppressor promoter control [185], cBAF-mediated oncogenic enhancer activation [116] and ncBAF-mediated CTCF-dependent remodelling of chromatin loop anchor activity [9,10,186]. For the latter, on the one hand, the loss of CTCF loop anchoring activity will disrupt boundary maintenance through the apparition of longer chromatin loops that enable ectopic enhancer hijacking that activates proto-oncogene promoters [187,188]. Conversely, increased loop anchoring activity will result in shorter chromatin loops that may lower tumour suppressor gene promoter activity. Redistributed ncBAF complexes may also relocalise to CTCF sites that control oncogenic super-enhancers [9,185,189], establishing pathological chromatin loops encompassing distal enhancers [190] that drive oncogenic gene expression, while also creating therapeutic vulnerability to ncBAF-specific inhibitors like BRD9 degraders [9].

## 5. Conclusions

The SMARCD family of SWI/SNF chromatin remodelling subunits represents a node where developmental programs, metabolic regulation and oncogenic transformation converge. SMARCD1, SMARCD2 and SMARCD3 exhibit tissue and cell state-specific activities and functional specialisation through selective incorporation into distinct SWI/SNF complex subtypes, recruitment by lineage-restricted transcription factors via their YEATS-like SWIFT domain [31] and post-translational modification by intracellular signal transduction cascades. Notably, SMARCD1’s exclusive incorporation into ncBAF complexes positions it uniquely at CTCF-dependent chromatin loop anchors, distinguishing it from its paralogs and potentially explaining its prominent oncogenic potential.

SMARCD subunits display profound context-dependent roles in cancer. While capable of enforcing differentiation and apoptotic programs, they predominantly function as oncogenic drivers when dysregulated through the loss of tumour-suppressive microRNAs and genomic alterations. Mechanistically, SMARCD-driven oncogenesis appears to operate through multiple convergent pathways, including corruption of WNT signalling, immune checkpoint evasion, metabolic reprogramming and oncogenic transcription factor recruitment. The disruption of the balance between PBAF, cBAF and ncBAF complexes in cancer is likely to enable enhancer hijacking, where ncBAF redistribution to altered CTCF sites grants oncogenic enhancers ectopic access to proto-oncogene promoters.

Critical knowledge gaps remain, including mechanisms governing paralog-selective complex assembly and post-translational modification of SWI/SNF subunits and the actual role of ncBAF at CTCF-dependent chromatin loop anchors. Addressing these will require structural characterisation of SMARCD-chromatin interactions, mapping of chromatin loop rewiring in SMARCD1 mutant cancers to validate the enhancer hijacking model and defining how complex composition varies across tumour heterogeneity. Therapeutically, the differential incorporation of SMARCD1 into ncBAF suggests BRD9 degraders may selectively target SMARCD1-driven malignancies, while SMARCD2/3-driven cancers may require alternative approaches targeting cBAF/PBAF functions. Ultimately, understanding how SMARCD subunits translate cellular context into distinct chromatin states will enable therapies that exploit oncogenic dependencies while sparing healthy tissue functions.

## Data Availability

No new data were created or analysed in this study. Data sharing is not applicable to this article.

## Appendix A

**Table A1:** Annotated features and post-translational modifications of SMARCD1, SMARCD2 and SMARCD3. Residue positions are given as amino acid numbers from the aligned coordinates. Colour codes correspond to annotation categories used in sequence visualisation (Figure A1).

Description	Colour	SMARCD1 Position	SMARCD2 Position	SMARCD3 Position	Database	Source	Identification Method
Disordered		1–119	215–236	72–149	Uniprot		
Gly residues		18–27			Uniprot		
Interaction with ESR1, NR1H4, NR3C1, PGR and SMARCA4		53–189				[191]	Functional
Interaction with SMARCC1 and SMARCC2		190–507				[191]	Functional
Necessary for GR/NR3C1-mediated remodelling and transcription from chromatin; required for GR/NR3C1 interaction with the BRG1/SMARCA4 complex in vivo		202–548			Uniprot	[191]	Functional
Interaction with UNK-C-ter; necessary for Rac/unkempt-dependent ubiquitination				437–498		[192]	Functional
SWIB/MDM2 domain		323–400	323–400	323–400	PROSITE		
Coiled coil		445–473	168–187; 421–477; 468–548		Uniprot	[63]	Functional
YEATS-like SWIFT domain (two parallel four-stranded β-sheets)		199–213; 239–248; 270–277; 288–292; 302–308; 313–321; 404–410; 420–430	114–138; 164–173; 188–195; 206–210; 220–226; 231–239; 329–335; 345–355	199–213; 239–248; 270–276; 288–292; 302–308; 313–321; 404–410; 420–430			Functional
Initiator methionine removed				17	Uniprot	[193]	HTP-MS
N-acetylalanine				18	Uniprot	[193]	HTP-MS
Mono-methylation		6; 43; 73; 104; 116; 117	4; 118; 546	118	Uniprot; PhosphoSitePlus	[194,195,196]	HTP-MS
Di-methylation		104; 116	73; 81; 104; 116		PhosphoSitePlus	[196,197]	HTP-MS
Asymmetric dimethylarginine		81; 104	81; 104		Uniprot	[196]	HTP-MS
Glycyl lysine isopeptide (Lys-Gly) (interchain with G-Cter in SUMO2)		117	236		Uniprot	[198]	HTP-MS
Sumoylated lysine		117; 153; 178; 256; 468; 497	153; 178; 216; 236; 247; 261; 306; 314; 418; 456; 497	57; 153; 178; 247; 261; 306; 386; 482; 497	PTMeXchange	[198,199,200,201,202,203]	HTP-MS
Ubiquitinated lysine		117; 178; 195; 216; 256; 261; 265; 271; 279; 306; 374; 386; 418; 419; 468; 497; 531	153; 178; 195; 216; 236; 247; 261; 263; 265; 271; 279; 306; 314; 329; 393; 418; 419; 456; 468; 497; 531	178; 247; 261; 263; 265; 271; 279; 374; 482; 497	PTMeXchange; PhosphoSitePlus	[198,199,200,201,202,203,204,205,206,207,208,209,210,211,212]	HTP-MS
Acetyllysine		153; 256	153; 393; 531	18; 153	PTMeXchange; PhosphoSitePlus	[198,213]	HTP-MS
Phosphothreonine		37; 83; 236	211; 224; 227; 231; 342; 344; 479; 500	137; 211; 294; 342	PRIDE; Uniprot; PhosphoSitePlus	[205,214,215,216,217,218,219,220,221,222,223,224]	HTP-MS
Phosphotyrosine		59; 97; 169	169; 207; 355; 369	169	PhosphoSitePlus	[225,226,227]	HTP-MS
Phosphoserine		217	15; 209; 213; 215; 221; 476; 478	209; 233; 236; 239; 312; 476	PRIDE; Uniprot	[74,205,218,220,221,222,223,224,227,228,229,230,231]	Functional [74]; HTP-MS

## Appendix B

**Table A2:** SMARCD paralog involvement in different cancers. For each entry, the cancer type, proposed functional role and reported transcription factor interactors are indicated.

Paralog	Cancer Type	Role in Cancer	Interactors/TFs	Ref.	Year	First Author
SMARCD1	Prostate cancer	Oncogene	AR	[139]	2009	Van De Wijngaart et al.
SMARCD1	Prostate cancer	Oncogene	—	[144]	2011	Sun et al.
SMARCD1	Prostate cancer (CSCs)	Oncogene	GR	[145]	2016	Rane et al.
SMARCD1	Neuroendocrine prostate cancer	Oncogene	E2F1	[180]	2021	Hagiwara et al.
SMARCD1	Prostate cancer	Oncogene	AR	[133]	2022	Ertl et al.
SMARCD1	Prostate cancer (enhancer-addicted)	Oncogene	AR	[146]	2022	Xiao et al.
SMARCD1	Prostate cancer (CRPC)	Oncogene	AR	[145]	2023	Waseem et al.
SMARCD1	Prostate cancer	Oncogene	HOXB13; AR	[175]	2025	Ersoy-Fazlioglu et al.
SMARCD1	Breast cancer	Oncogene	—	[151]	2012	Stephens et al.
SMARCD1	Breast cancer (BCSC)	Oncogene	—	[147]	2014	Deng et al.
SMARCD1	Breast cancer	Oncogene	—	[150]	2017	Liu & Ye
SMARCD1	Breast cancer (ER+/HER2-enriched)	Context-dependent	—	[181]	2024	Ross et al.
SMARCD1	Hepatocellular carcinoma	Oncogene	—	[169]	2019	Xiong et al.
SMARCD1	Hepatocellular carcinoma	Oncogene	—	[167]	2020	Zhou et al.
SMARCD1	Hepatocellular carcinoma	Oncogene	—	[168]	2022	Fan et al.
SMARCD1	Hepatocellular carcinoma	Oncogene	—	[172]	2024	Li et al.
SMARCD1	Gastric cancer	Oncogene	—	[149]	2015	Shen et al.
SMARCD1	Gastric cancer (GCSCs)	Oncogene	—	[159]	2018	Akrami et al.
SMARCD1	Gastric cancer (GCSCs)	Oncogene	—	[160]	2025	Akrami et al.
SMARCD1	Ovarian cancer	Oncogene	—	[182]	2010	Meunier et al.
SMARCD1	Ovarian cancer (serous)	Oncogene	—	[183]	2017	Arts et al.
SMARCD1	Ovarian cancer	Oncogene	—	[184]	2017	Wang et al.
SMARCD1	Acute myeloid leukaemia	Oncogene	—	[176]	2020	Rashid et al.
SMARCD1	Bladder cancer	Oncogene	—	[148]	2022	Tamai et al.
SMARCD1	Colorectal cancer	Oncogene	—	[161]	2025	Fu et al.
SMARCD1	Diffuse large B-cell lymphoma	Oncogene	—	[163]	2024	Nakade et al.
SMARCD1	Gestational choriocarcinoma	Oncogene	—	[152]	2020	Jung et al.
SMARCD1	Glioblastoma	Tumour suppressive	Notch1; p53	[142]	2021	Zhu et al.
SMARCD1	Skin cutaneous melanoma	Oncogene	—	[140]	2023	Chen et al.
SMARCD1	Oesophageal squamous cell carcinoma	Oncogene	—	[162]	2025	Wang et al.
SMARCD1	Oral squamous cell carcinoma	Oncogene	mutant p53	[29]	2020	Adduri et al.
SMARCD1	Small cell lung cancer (POU2F3+)	Oncogene	POU2F3	[30]	2024	Duplaquet et al.
SMARCD2	MLL-rearranged leukaemia	Oncogene	—	[178]	2015	Cruickshank et al.
SMARCD2	Diffuse large B-cell lymphoma	Oncogene	—	[163]	2024	Nakade et al.
SMARCD2	Paediatric undifferentiated carcinoma	Tumour suppressive	—	[179]	2021	Stirnweiss et al.
SMARCD3	Breast cancer	Oncogene	—	[153]	2013	Jordan et al.
SMARCD3	Breast cancer	Oncogene	—	[154]	2016	Gregoire et al.
SMARCD3	ER+ Breast cancer	Oncogene	—	[173]	2021	Tropée et al.
SMARCD3	ER+ Breast cancer (everolimus-resistant)	Oncogene	—	[174]	2025	Medina et al.
SMARCD3	Gastric cancer	Oncogene	—	[155]	2024	Park SY et al.
SMARCD3	Gastric cancer	Oncogene	—	[156]	2025	Park JH et al.
SMARCD3	Cervical cancer	Tumour suppressive	—	[138]	2024	An et al.
SMARCD3	Colorectal cancer (CAFs)	Oncogene	—	[157]	2020	Jiang et al.
SMARCD3	Endometrial cancer	Oncogene	—	[165]	2023	Pang et al.
SMARCD3	Medulloblastoma	Oncogene	—	[158]	2023	Zou et al.
SMARCD3	Neuroblastoma	Oncogene	—	[166]	2004	Takita et al.
SMARCD3	Pan-cancer	Oncogene	—	[164]	2024	Guo et al.
SMARCD3	Pancreatic ductal adenocarcinoma	Oncogene	FOXA1	[139]	2023	Ferguson et al.
SMARCD3	Prostate cancer	Tumour suppressive	—	[34]	2025	Ertl et al.
